# Combining variant detection and fragment length analysis improves detection of minimal residual disease in postsurgery circulating tumour DNA of stage II–IIIA NSCLC patients

**DOI:** 10.1002/1878-0261.13267

**Published:** 2022-06-27

**Authors:** Daan C. L. Vessies, Milou M. F. Schuurbiers, Vincent van der Noort, Irene Schouten, Theodora C. Linders, Mirthe Lanfermeijer, Kalpana L. Ramkisoensing, Koen J. Hartemink, Kim Monkhorst, Michel M. van den Heuvel, Daan van den Broek

**Affiliations:** ^1^ Department of Laboratory Medicine Netherlands Cancer Institute – Antoni van Leeuwenhoek Hospital Amsterdam The Netherlands; ^2^ Department of Pulmonology Radboud University Medical Center Nijmegen The Netherlands; ^3^ Biometrics Department Netherlands Cancer Institute – Antoni van Leeuwenhoek Hospital Amsterdam The Netherlands; ^4^ Department of Thoracic Oncology Netherlands Cancer Institute – Antoni van Leeuwenhoek Hospital Amsterdam The Netherlands; ^5^ Department of Surgery Netherlands Cancer Institute – Antoni van Leeuwenhoek Hospital Amsterdam The Netherlands; ^6^ Department of Pathology Netherlands Cancer Institute – Antoni van Leeuwenhoek Hospital Amsterdam The Netherlands

**Keywords:** circulating cell‐free tumour DNA, ctDNA, fragmentomics, minimal residual disease, MRD, NSCLC

## Abstract

Stage II–IIIA nonsmall cell lung cancer (NSCLC) patients receive adjuvant chemotherapy after surgery as standard‐of‐care treatment, even though only approximately 5.8% of patients will benefit. Identifying patients with minimal residual disease (MRD) after surgery using tissue‐informed testing of postoperative plasma circulating cell‐free tumour DNA (ctDNA) may allow adjuvant therapy to be withheld from patients without MRD. However, the detection of MRD in the postoperative setting is challenging, and more sensitive methods are urgently needed. We developed a method that combines variant calling and a novel ctDNA fragment length analysis using hybrid capture sequencing data. Among 36 stage II–IIIA NSCLC patients, this method distinguished patients with and without recurrence of disease in a 20 times repeated 10‐fold cross validation with 75% accuracy (*P* = 0.0029). In contrast, using only variant calling or only fragment length analysis, no signification distinction between patients was shown (*P* = 0.24 and *P* = 0.074 respectively). In addition, a variant‐level fragmentation score was developed that was able to classify variants detected in plasma cfDNA into tumour‐derived or white‐blood‐cell‐derived variants with 84% accuracy. The findings in this study may help drive the integration of various types of information from the same data, eventually leading to cheaper and more sensitive techniques to be used in this challenging clinical setting.

## Graphical abstract

Blood and tissue from 36 stage II–III NSCLC patients and 15 risk‐ and age‐matched patients with nonmalignant disease were analysed with targeted hybrid capture sequencing to detect postsurgery minimal residual disease (MRD) in the blood, prior to adjuvant therapy. Our combined variant calling and fragmentomics MRD model was capable of stratifying patients with and without recurrence with 75% accuracy (*P* = 0.0029).

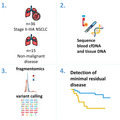

**Abbreviations**
BCP: blood cell pellet
cfDNA: circulating cell‐free DNA
CHIP: clonal haematopoiesis of indeterminate potential
ctDNA: circulating cell‐free tumour DNA
DFS: disease‐free survival
FS: fragmentation score
IQR: inter‐quartile range
LACE: lung adjuvant cisplatin evaluation
LEMA: lung early molecular assessment (trial)
METC: medical ethics committee
MRD: minimal residual disease
NKI: Nederlands Kanker Instituut (Netherlands Cancer Institute)
NSCLC: nonsmall cell lung cancer
TTF‐CV: twenty times repeated 10‐fold cross validation
VFS: variant‐level fragmentation score
WGS: whole‐genome sequencing

## 1 Introduction

Lung cancer is the leading cause of cancer‐related deaths worldwide [1]. At diagnosis, 40–50% of nonsmall cell lung cancer (NSCLC) patients present with stage I–III disease [2, 3]. Resection is the primary treatment approach for stage I–II disease, and an important component of the multimodality approach for stage III. Based on a meta‐analysis of multiple randomised controlled trials the standard of care for stage II–IIIA NSCLC includes adjuvant chemotherapy, even though the absolute disease‐free survival (DFS) benefit is limited (5.8%) [4, 5]. Moreover, adjuvant personalised regimens have been registered recently, using targeted therapy or immune checkpoint inhibitors [6, 7]. Consequently, there is an unmet clinical need to identify patients who will not benefit from adjuvant therapy.

The prospect of using circulating cell‐free DNA (cfDNA) to detect postoperative minimal residual disease (MRD) was met with initial optimism [8, 9]. However, although early detection of relapse using cfDNA has been reported in gastric cancer [10] and colon cancer [11], as well as for NSCLC post‐therapy [12, 13], cfDNA as a postoperative marker to identify NSCLC patients who will not benefit from adjuvant therapy is not yet reported.

Traditionally the detection of MRD is focussed on detecting somatic variants in the resected tissue material, and tracing those in the postoperative or postadjuvant therapy plasma [8, 9, 10, 11, 12, 13, 14]. Approaches using the same panel for all patients can be limited by the number of variants that are available for tracking. To overcome this, tissue‐informed personalised assays have been developed, tailored to every individual patient, to trace up to 48 mutations in plasma [12, 13, 14, 15]. However, designing and analysing such individualised assays is costly and time‐consuming, which may be problematic in between surgery and adjuvant therapy.

More recently, other approaches have been developed using additional characteristics of cfDNA, next to mutations, to help detect the presence of circulating tumour DNA (ctDNA). One promising approach is the interrogation of cfDNA fragment length, leveraging the knowledge that ctDNA is shorter than nontumour‐derived cfDNA [16, 17, 18, 19, 20, 21]. This has been used to infer a patient‐level fragmentation‐based classifier from shallow whole‐genome sequencing (WGS) data [22, 23], as well as to help distinguish tumour‐derived mutation calls from clonal haematopoiesis‐derived mutation calls in hybrid capture sequencing data [19, 24, 25]. This mounting evidence suggests that fragment length analysis could also be used to support the classical variant‐based detection of MRD. As fragment length analysis and variant tracing are independent read‐outs of the presence of ctDNA, there is an opportunity to combine the two approaches to improve the sensitivity of detecting MRD.

In this proof‐of‐principle study we explore the potential of combining patient‐level fragment length analysis and variant calling from hybrid capture sequencing data for MRD detection in stage II–IIIA NSCLC patients.

## 2 Materials and methods

**Fig. 1:**
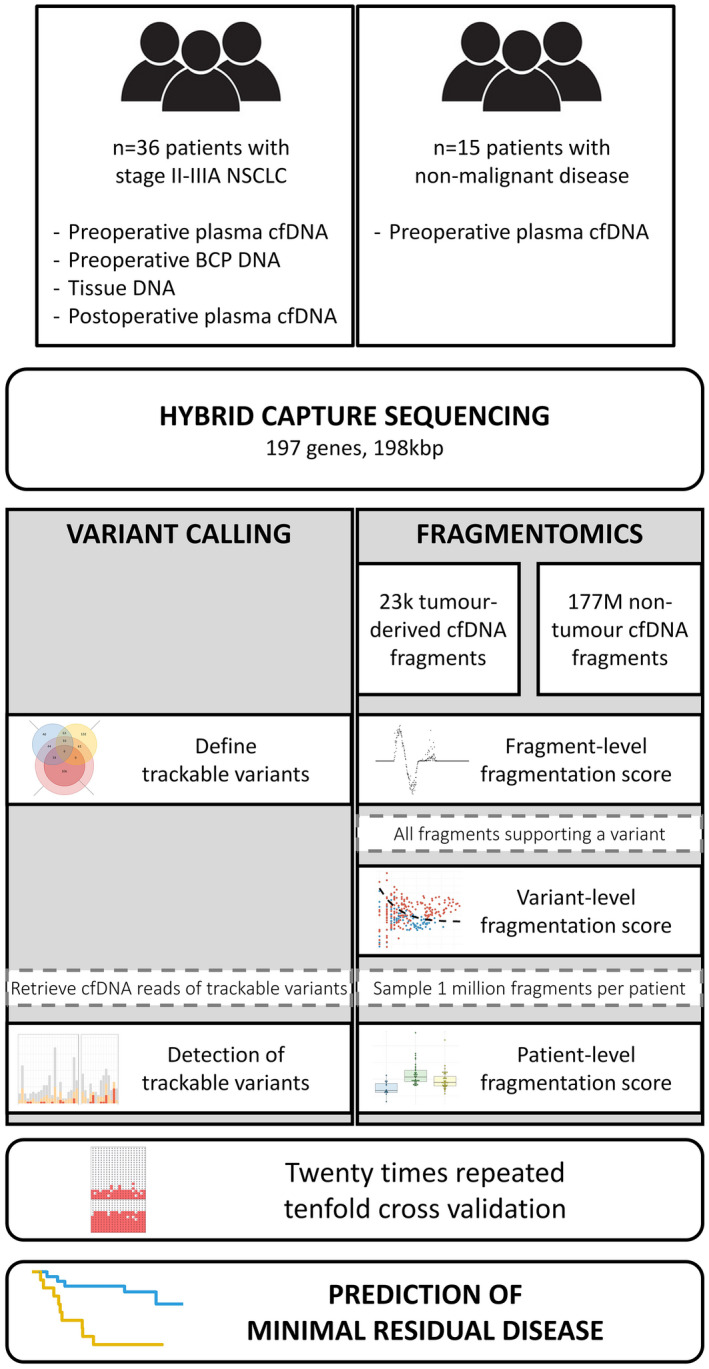
Flow chart illustrating the experimental procedures and data flows in this study. Blood and tissue samples from 36 NSCLC patients and plasma cfDNA from 15 risk‐ and age‐matched patients with nonmalignant disease were sequenced with a targeted hybrid capture sequencing panel. Optimised variant calls were combined with patient‐level fragmentomics, both from the hybrid capture sequencing data, to determine the presence of minimal residual disease in each patient in a 20 times repeated 10‐fold cross validation. [Colour figure can be viewed at wileyonlinelibrary.com]

A flow chart illustrating the procedures and data streams in this project is provided in Fig. 1.

### 2.1 Patients

All patients were enrolled with written informed consent as part of the multi‐centre Lung Early Molecular Assessment trial (LEMA; ClinicalTrials.gov NCT02894853), which was in accordance with the standards set in the declaration of Helsinki and was approved by the medical ethics committee (METC) of the Netherlands Cancer Institute (NKI). Patients were only included in this MRD study if they were diagnosed with pathological stage II–IIIA NSCLC disease and if resected tissue material, preoperative plasma (0–50 days prior to surgery), preoperative BCP and postoperative plasma were available. The postoperative plasma was required to be taken at least 3 days postsurgery [26], and before adjuvant therapy, with a maximum of 36 days postsurgery. A cohort of 36 patients meeting these criteria was selected.

Also, a control group was selected from the LEMA trial and consisted of 15 risk‐ and age‐matched patients with a suspicion of lung cancer based on imaging, who subsequently underwent a tissue biopsy which proved a nonmalignant diagnosis. This cohort of risk‐ and age‐matched controls is a reflection of daily clinical practice where we need to distinguish between patients with lung cancer and patients with nonmalignant diseases of the lung. Details of the nonmalignant control group are provided in Table S1. For the nonmalignant control group only the preoperative plasma sample was sequenced as described below.

### 2.2 Samples

Blood was collected in two hospitals, either using 10 mL K_2_‐EDTA tubes or 10 mL cell‐stabilising tubes (CST; STRECK, Omaha, NE, USA). Cell‐free plasma was obtained from the K_2_‐EDTA tube within 4 h by a two‐step centrifugation at room temperature: 20 min at 380 ***g*** followed by 10 min at 20 000 ***g***. Cell‐stabilising tubes were centrifuged at room temperature for 10 min at 1700 ***g*** and 10 min at 20 000 ***g*** within 7 days. Cell‐free plasma was stored in 1–4 mL aliquots at −80 °C. cfDNA isolation was performed using the QIAsymphony Circulating DNA kit (article number 1091063, Qiagen, Dusseldorf, Germany) with the QIAsymphony (Qiagen). No extraction blanks were used in this study. Elution volume was set to 60 μL and samples were stored at 4 °C until use. No significant differences were observed in the fragmentation scores of samples collected in EDTA tubes or CST (data not shown). To confirm this, a pilot experiment was performed with nine patients with metastatic NSCLC. Blood was concurrently drawn in both CST and EDTA tubes, and patient‐level FS was determined. Based on a Passing Bablok regression we conclude that the type of tube does not influence the patient‐level FS (Fig. S1B).

DNA from BCP was isolated from a 1 mL pellet using the QIAsymphony DSP DNA Midi Kit (article number 937255, Qiagen). Elution volume was set to 400 μL and samples were stored at 4 °C until use. DNA was fragmented sonically on a Covaris ME220 Focused‐ultrasonicator (Covaris Inc., Woburn, MA, USA) using microTUBE AFA Fibre Pre‐Slit Snap‐Cap (PN 520045) vessels, with the following settings: Duration 70 s, Peak Power 70 W, Duty Factor 20% and 1000 Cycles per Burst.

DNA from tissue was obtained from FFPE slides. The pathologist scored tumour percentage and indicated most tumour‐dense region for isolation on an H&E slide. Five to 10 (depending on tumour size) FFPE 10 μm slides were used. DNA and RNA were isolated simultaneously with the AllPrep DNA/RNA FFPE isolation kit (Qiagen, #80234) by using the QIAcube, according to the manufacturer's protocol. DNA input into the AVENIO library preparation phase was determined according to the protocol (median 37.3 ng, IQR 32.6–46.3 ng). Fragmentation of the FFPE tissue DNA was performed enzymatically, according to the AVENIO library preparation protocol.

### 2.3 Sequencing and variant calling

Fourteen preoperative samples were sequenced with a large capture panel comprising 1.1 Mb as described earlier [27], which fully overlaps the AVENIO Surveillance Panel and only the overlapping regions were used. All other samples (22/36 preoperative plasma, BCP, tissue and postoperative plasma of 36 NSCLC patients, as well as the preoperative plasma samples of patients with nonmalignant disease) were sequenced in‐house using the AVENIO Surveillance Panel (for Research Use Only; not for use in diagnostic procedures, Roche Sequencing Systems, Inc. Pleasanton, CA, USA), covering hypermutated regions or full exonic sequences of 197 genes, total size 198 kb [28]. Handling in accordance with the predefined protocol, we isolated cfDNA from all available plasma and used 50 μL of the eluate as input for the AVENIO library preparation. Median cfDNA input for preoperative samples was 24.4 ng (IQR: 17.4–38.5 ng), for postoperative samples was 50.0 ng (IQR: 49.4–50.0 ng).

Samples were multiplexed and sequenced on an Illumina NextSeq550, generating median 30 m reads per sample (IQR: 27–34 m). Median unique sequencing depth in preoperative samples was 3678× (IQR: 2495–4758×), in postoperative samples 6289× (IQR: 5081–6980×), in BCP 3428× (IQR: 3162–3684×) and in tissue 1938× (IQR: 1573–2819×).

Variant calling was performed using the AVENIO pipeline, using the unfiltered called variants. All variants that were detected in blood cell pellet were considered to be germline if they were also detected in tumour tissue. They were considered to be CHIP if they were not detected in tumour tissue. Germline variants and CHIPs were removed from downstream analysis. All variants except germline variants are reported in Table S2. Raw data read counts were extracted from the .freq files of the postoperative plasma for all variants detected in any sample of that patient.

Variant calling cut‐offs in the postoperative plasma were optimised. Specifically, the cut‐offs were lowered for tumour‐informed and preoperative plasma‐informed variants. We iteratively lowered the cut‐offs to requiring one to eight reads. Additionally, the cut‐off for calling a patient MRD‐positive varied from requiring at least one to six baseline‐informed variants detected in the postoperative plasma. The best combination of cut‐offs was selected based on the highest concordance with recurrence status of the patients.

### 2.4 Fragmentation score

To calculate the FS, we first built a reference database of reads that contained tumour‐informed mutations, and stored their respective lengths from the deduplicated BAM files, in total 21 705 fragments. For the nontumour reads we collected reads from 15 patients with nonmalignant disease (177.6 million fragments).

**Fig. 2:**
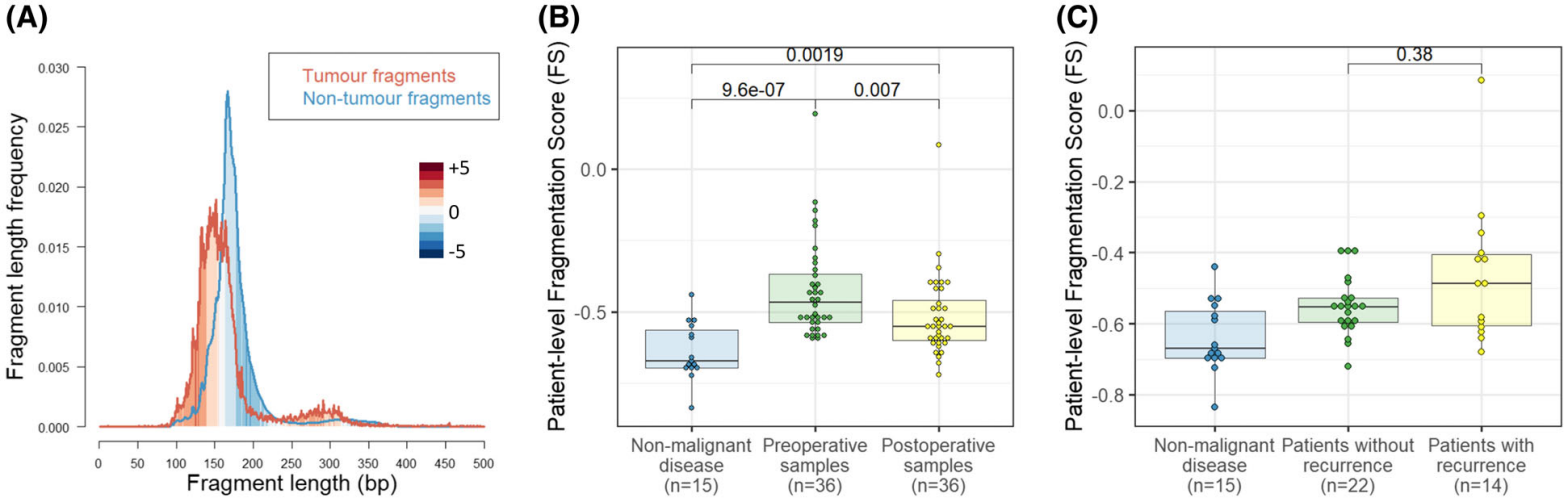
Fragment length analysis. (A) Fragment length density in the perspective of fragment length in base pairs (bp) including tumour fragments (red, *n* = 21 705 fragments), defined by containing a tumour‐derived mutation, and nontumour fragments (blue, *n* = 177.6 million fragments) from patients with nonmalignant disease. Red shaded areas indicate fragment lengths that are more prevalent in tumour cfDNA, while blue shaded areas are more prevalent in nontumour cfDNA. The intensity of colours corresponds to the log‐2 of the relative ratio of tumour‐ to nontumour‐derived fragments. (B) Patient‐level fragmentation score (FS) for age‐matched nonmalignant patients (i.e. control group, *n* = 15), and paired preoperative and postoperative plasma samples from NSCLC patients (*n* = 36). Fragmentation score was significantly higher in both preoperative patient samples and postoperative patient samples when compared to nonmalignant patients (*P* < 0.001 and *P* = 0.002 respectively, Wilcoxon rank sum test). FS was significantly higher in preoperative patient samples compared to paired postoperative patient samples (*P* = 0.007, paired *t*‐test). (C) Patient‐level FS for age‐matched nonmalignant patients (i.e. control group, *n* = 15), and postoperative plasma samples from NSCLC patients, categorised in patients with (*n* = 14) versus without recurrence of disease (*n* = 22). There was no statistically significant difference between the postoperative FS of patients with recurrence and patients without recurrence of disease (*P* = 0.38, Wilcoxon rank sum test). [Colour figure can be viewed at wileyonlinelibrary.com]

First, we randomly sampled 10 000 reads from each set and calculated the probability density for each fragment length to occur in tumour‐ and nontumour‐derived cfDNA (Fig. 2A). Next, we calculated the log‐2 of the ratio of these densities, maxed at +5 and −5 for lengths that had a count of 0 in either group. Additionally, fragment lengths that had a total of 20 reads or fewer were given a score of 0. This process was bootstrapped over 1000 iterations to smooth out any noisy areas and reduce the impact of sampling errors.

Thus, each fragment length was allocated a per‐fragment fragmentation score in this reference set, illustrated in Fig. S2A. In order to translate this per‐fragment score into a per‐patient score, we randomly sampled 1 million fragments from each patient and reported the mean fragmentation score per million fragments (Fig. 2A,B and Fig. S3A). Patients who had an FS greater than the mean FS plus two times the standard deviation among 15 plasma samples from patients with nonmalignant disease were considered positive for MRD detection. Technical reproducibility of the FS was shown by 10 times repeated subsampling of one million, hundred thousand, ten thousand or one thousand reads per sample (Fig. S4A). The confidence interval of the calculated FS was consistently smaller than 0.01 when one million reads were sampled, indicating very consistent patient‐level FS reproducibility at this sampling size (Fig. S4B).

**Fig. 3:**
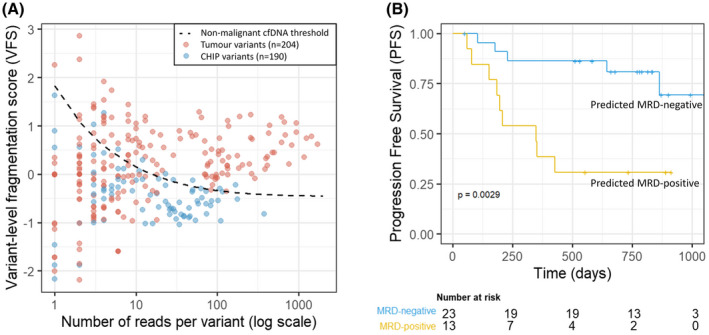
Variant level fragment length analysis and Kaplan–Meier curve of the combined MRD‐model. (A) Variant‐level fragmentation score (VFS) versus number of reads per variant for all tumour‐informed variants (*n* = 204, red) and CHIP variants (*n* = 190, blue) in the LEMA‐MRD cohort. Reads from preoperative and postoperative plasma for the same variant were added up for this analysis. The black dashed line represents the mean plus two times standard deviation of fragments randomly sampled from 15 patients with nonmalignant disease and is used as a nonmalignant cfDNA threshold. (B) Performance of a combined MRD model including fragment length analysis and variant calling. Kaplan–Meier curve with progression‐free survival (PFS) of MRD‐positive (yellow) and MRD‐negative patients (blue) based on the combined variant calling and patient‐level fragmentation score (FS) model in a 20 times repeated 10‐fold cross validation. Patients were labelled as MRD‐positive or ‐negative by the majority result of the cross validation. The model was able to differentiate between patients with recurrence of disease and those without (*P* = 0.0029, log‐rank test). [Colour figure can be viewed at wileyonlinelibrary.com]

The per‐fragment score was translated to a per‐variant score (VFS) by averaging the scores of all fragments supporting a specific variant (Fig. 3A). If the same variant was detected in both the preoperative and postoperative plasma, the fragments were analysed collectively in order to obtain more fragments per variant, resulting in a better score. The nonmalignant cfDNA threshold was established by randomly sampling each number of reads from 15 patients with nonmalignant disease 1000 times, and calculating the VFS. The threshold was set at the mean plus two times the standard deviation in nonmalignant reads. The minimum number of reads to include a variant was determined by assessing the best performance by assigning a score of 1 for each correctly classified variant, −1 for incorrectly classified variants and 0 for indeterminate variants below the cut‐off, and resulted in a cut‐off of eight reads. The same cut‐offs were applied to the validation data in the MSKCC/Grail cohort (Fig. S3B).

The algorithm to calculate the FS and VFS was written in R [29] and has been made publicly available (https://github.com/DCLVessies/Fragmentomics) for other researchers to evaluate, along with the established fragment length reference set. The FS and VFS presented in this work could in principle be used in any cfDNA sequencing method that preserves the fragment length information, such as hybrid capture sequencing and (shallow) whole‐genome sequencing, but not in PCR‐based amplicon sequencing.

### 2.5 Cross validation

The applicability of the combined variant calling and FS model for predicting recurrence was validated using a 20 times repeated 10‐fold cross validation. For each repeat, the 36 patients were randomly divided into 10 folds of three or four patients. In each iteration the model was trained on nine folds, and the training algorithm was applied on the remaining fold until each fold had been applied once. In total, this process of cross validation was repeated 20 times.

For each fold, the reference set for the FS was rebuilt using only the mutation reads in the 90% of the data used for training, and all FS including the FS for patients with nonmalignant disease were recalculated. Cut‐offs for the variant calling were determined likewise based on the training data. The variant calling and FS each provided a true or false call based on their respective cut‐offs as described above. Based on the best fit of the training data to the status of disease recurrence, the model determined whether both outcomes had to be positive or whether one positive outcome was sufficient. Subsequently, this algorithm was applied to the one remaining fold that was not included in the training data. The final performance of the model was determined by the majority call for each patient among the 20 repeats – that is, a patient was counted as predicted positive if it was predicted positive at least 11 times (Fig. S5).

For the randomly assigned patient‐level FS as shown in Fig. S6C, the FS and variant calls were determined as described above, but subsequently, the patient‐level FS was assigned to a randomly determined other NSCLC patient.

### 2.6 Potential clinical implications

Based on the Lung Adjuvant Cisplatin Evaluation (LACE) meta‐analysis of five randomised controlled trials [4], adjuvant chemotherapy after complete resection of stage II and III NSCLC was established as the standard of care [5]. The absolute disease‐free survival benefit of adjuvant chemotherapy was determined to be 5.8% in the meta‐analysis, and this was assumed to be the case in our simulations.

In order to estimate the fraction of patients that benefit from adjuvant chemotherapy in the MRD‐positive and ‐negative groups, we assumed that the sensitivity of detecting recurrence is the same as the sensitivity for detecting patients who would benefit from chemotherapy. While this assumption is not ideal, this is the closest estimate we have based on the data generated in this study.

In a 10 000 times repeated bootstrap simulation 36 patients were randomly drawn from our cohort, with replacement. Next, each of the 36 patients was randomly assigned a prediction: MRD‐positive or MRD‐negative with probability equal to the results of the TTF‐CV (Fig. S5, rightmost column). In each iteration the 5.8% of people who benefit from adjuvant chemotherapy were distributed between the MRD‐negative and ‐positive groups proportionally to the sensitivity for detecting recurrence in that iteration (e.g. if sensitivity for detecting recurrence was 80% in that iteration, then likewise 80% of the 5.8% of patients who would benefit from adjuvant chemotherapy were allocated to the MRD‐positive group). The fraction of MRD‐positive and ‐negative patients who would benefit from adjuvant chemotherapy was reported.

## 3 Results

In total 36 stage II–IIIA NSCLC patients with available preoperative blood cell pellet (BCP) and plasma, resected tissue material and postoperative plasma between 3 and 36 days postsurgery were included in this study. In addition, 15 patients with nonmalignant disease and with available preoperative blood plasma were selected as a control group. All patients were selected from the larger Lung Early Molecular Assessment trial (LEMA; ClinicalTrials.gov NCT02894853).

### 3.1 Patient characteristics

In this cohort six patients (17%) were diagnosed with pathological stage IIA, 18 patients (50%) with stage IIB and 12 patients (33%) with stage IIIA disease. In total, recurrence of disease occurred in 14 patients (39%) with a median follow‐up of 23 months (IQR 19–30 months). The clinical characteristics of the assessed cohort are represented in Table 1. A total of 16 patients (44%) had squamous cell carcinoma, in line with national prevalence of this histological subtype in stage II (35%) and III (36%) NSCLC patients in the Netherlands [30].

**Table 1:** Clinical characteristics of NSCLC patients in this cohort. LCNEC, Large‐cell neuroendocrine carcinoma; NSCLC‐NOS, Nonsmall cell lung cancer – not otherwise specified.

	All patients	Stage II	Stage III
	*N* = 36	*N* = 24	*N* = 12
Age, median years (IQR)	68 (62–76)	69 (62–75)	67 (61–76)
Sex, *n* (%)			
Male	23 (64)	15 (63)	8 (67)
Female	13 (36)	9 (37)	4 (33)
Smoking status, *n* (%)			
Active	11 (31)	8 (33)	3 (25)
Former	25 (69)	16 (67)	9 (75)
Pack years, median (IQR)	38 (20–57)	37 (20–55)	43 (23–70)
Tumour histology, *n* (%)			
Adenocarcinoma	18 (50)	13 (54)	5 (42)
Squamous cell carcinoma	16 (44)	10 (42)	6 (50)
NSCLC‐NOS	1 (3)	1 (4)	0
LCNEC	1 (3)	0	1 (8)
Recurrence of disease, *n* (%)*			
Yes	14 (39)	7 (29)	7 (58)
No	22 (61)	17 (71)	5 (42)
Adjuvant chemotherapy			
Yes, completed	7 (19)	5 (21)	2 (17)
Yes, partially completed	7 (19)	3 (12)	4 (33)
No	22 (61)	16 (67)	6 (50)
Days between baseline plasma and surgery, median (IQR)	8 (6–14)	8 (6–14)	9 (6–15)
Days between surgery and postoperative plasma, median (IQR)	10 (6–23)	13 (6–25)	6 (4–20)
Months follow‐up, median (IQR)	23 (19–30)	26 (22–30)	19 (7–23)

### 3.2 Somatic variants

**Fig. 4:**
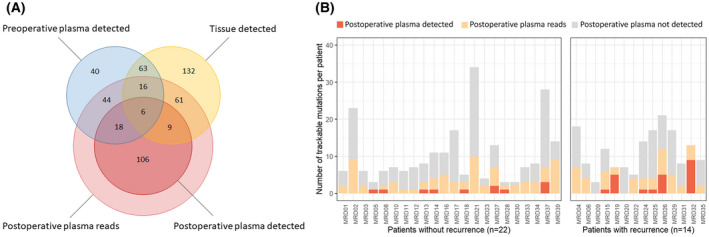
Variants detected in tumour tissue and pre‐ and postoperative plasma. (A) Total number of absolute variants across 36 patients detected in tumour tissue (yellow), preoperative plasma (blue) and/or postoperative plasma (red), including a differentiation in variants called by the Avenio pipeline or only with supporting reads. (B) Number of trackable variants per patient that were detected or had supporting reads in the postoperative plasma, categorised in patients with (*n* = 14) versus patients without recurrence of disease (*n* = 22). Trackable variants are defined as detected variants in the preoperative setting in either plasma or tumour tissue. Trackable variants in dark orange were detected by the Avenio pipeline, trackable variants in light orange were not detected by Avenio but did have reads in the alignment files. Trackable variants in grey did not have reads in the postoperative plasma alignment files. [Colour figure can be viewed at wileyonlinelibrary.com]

To detect MRD using variant calling, we first identified tumour‐related variants in the preoperative setting using tissue and plasma and subsequently sought whether these variants could be traced in the postoperative plasma. After removing clonal haematopoiesis of indeterminate potential (CHIP) and germline variants, a median of 8 (range 3–34) tissue‐informed or preoperative plasma‐informed variants per patient remained that could be tracked in the postoperative plasma. As such, variants that were only detected in postoperative plasma were considered uninformative and were removed from analysis. In total, 389 trackable variants were identified in 36 patients, of which 154 variants (40%) in the postoperative plasma were directly reported by the AVENIO pipeline or had reads in the deduplicated BAM files (Fig. 4A).

When considering the prognostic power of MRD detection using only variants, a median of 3 variants per patient (range 1–10) were detected or had reads in the postoperative plasma of patients who did not develop recurrence, in comparison to a median of 4 variants (range 0–13) in patients who did develop recurrence (Fig. 4B). Defining disease recurrence as a surrogate endpoint for the presence of MRD postsurgery, we performed a 20 times repeated 10‐fold cross validation (TTF‐CV, described in Materials and methods) to evaluate the performance of variant calling for detecting MRD. Using only variant calling with optimised variant call thresholds we were unable to accurately distinguish patients with and without recurrence (*P* = 0.24, log‐rank test, Fig. S6A).

### 3.3 Fragment length analysis

We investigated differences between ctDNA and nontumour cfDNA fragment lengths. ctDNA fragments were defined by containing a tumour tissue‐informed mutation, of which 21 705 fragments were detected in the plasma of 36 patients. In line with what others found, these ctDNA fragments were shorter than cfDNA fragments from patients with nonmalignant disease (control group), including both the mononucleosomal and the dinucleosomal fragments (Fig. 2A) [16, 18, 20]. Correspondingly, the relative abundance of each fragment length in ctDNA versus nontumour cfDNA fragments indicates the likelihood of each fragment originating from a tumour cell or a nontumour cell. As described in the Materials and methods section, this property was used to calculate an aggregated patient‐level Fragmentation Score (FS) from 1 million fragments per patient, derived from the same hybrid capture sequencing data as the variant calling.

Median patient‐level FS in the preoperative samples was −0.47 (IQR −0.54 to −0.37), which was higher than observed in the postoperative samples with a median of −0.55 (IQR −0.60 to −0.46, *P* = 0.007, paired *t*‐test, Fig. 2B). FS among 15 patients with nonmalignant disease (median −0.67, IQR −0.70 to −0.56) was lower than in preoperative samples (*P* < 0.001, Wilcoxon rank sum test) and in postoperative samples (*P* = 0.002, Wilcoxon rank sum test, Fig. 2B). Applying a cut‐off at the mean plus two times standard deviation of the patient‐level FS for patients with nonmalignant disease, we reached 100% specificity (95% CI 72%–100%), and sensitivity of 44% (95% CI 28%–62%) and 25% (95% CI 12%–42%) in preoperative and postoperative samples respectively. Subsequently, the performance of the patient‐level FS was validated in the DELFI cohort [22]. We applied a cut‐off of the mean plus two times standard deviation of the patient‐level FS among 213 samples from healthy individuals. This resulted in 98.6% specificity (95% CI 95.9%–99.7%) and 58% sensitivity among lung cancer cases (*n* = 12, 95% CI 28%–85%), confirming the performance of the patient‐level FS (Fig. S3A). However, the difference in postoperative FS between patients with and without recurrence was not significant (median −0.49 versus −0.55, *P* = 0.38, Wilcoxon rank sum test, Fig. 2C) and using only the FS we were unable to accurately distinguish patients with and without recurrence (*P* = 0.07, log‐rank test, Fig. S6B).

Additionally, a variant‐level fragmentation score (VFS) was developed to differentiate tumour‐informed variants from nontumour variants (e.g. CHIPs) based on the fragment length of the supporting reads. The VFS distinguished tumour‐informed variants from CHIPs with 84% specificity (159/190 CHIPs classified correctly) and 55% sensitivity (113/204 tumour variants classified correctly; Fig. 3A). When considering only variants with at least eight reads, the specificity was 82% (131/159 CHIPs) and sensitivity was improved to 86% (93/108 tumour variants). The performance of the VFS was validated in an independent cohort with high confidence calls of CHIPs and biopsy‐matched variants from Grail/MSKCC [31]. Using the exact same criteria and cut‐offs as established in our own cohort, we reached 93% specificity (106/114 CHIPs) and 82% sensitivity (263/319 tumour variants; Fig. S3B), confirming the robustness and generalisability of the VFS classification. However, including this classifier in the MRD detection model did not improve its ability to distinguish patients with and without recurrence since the variants were already correctly classified by having sequenced the tumour tissue and BCP.

### 3.4 Combined variants and FS model

To improve the accuracy of the MRD model we explored the possibility of combining the variant detection and FS approach. The method of combination and the TTF‐CV used to evaluate the performance of this combined model are described in Materials and methods. The clinical sensitivity of the combined variant calling and FS approach for detecting ctDNA in preoperative plasma was 75% (95% CI 58%–88%) at 100% specificity (95% CI 78%–100%), compared to 44% for FS alone (95% CI 28%–62%) and 47% for variant detection alone (95% CI 30%–64%), highlighting the complementarity of these approaches (Fig. S1A).

When applied to detect MRD, the combined model was able to differentiate patients with disease recurrence from those without with an accuracy of 75% (Fig. 3B, *P* = 0.0029, log‐rank test). The negative predictive value (NPV) was 78% (95% CI 56%–92%). The performance of this combined model was significantly superior in comparison to the model using only variant calling (Fig. S6A, *P* = 0.24, log‐rank test), only FS (Fig. S6B, *P* = 0.07, log‐rank test), or a model combining variant calling and randomly generated FS (Fig. S6C, *P* = 0.18, log‐rank test), indicating the addition of FS is truly informative.

Due to the small cohort size and limited number of events (*n* = 14), it was not possible to perform a multivariate Cox proportional hazards analysis. Instead, we evaluated the bivariate Cox hazard ratios of the MRD prediction model using each of the following factors as a covariate: disease stage (stage II versus stage IIIA), simultaneous secondary malignancies (yes versus no), completion of adjuvant chemotherapy (not started versus not completed versus completed) and the time between surgery and postoperative blood draw (in days). This revealed the MRD prediction model was a significant predictor of progression‐free survival (PFS) in all bivariate analyses, and only tumour stage was found to be a significant covariate (Table S3).

### 3.5 Potential clinical implications

To explore the potential effect of implementing an MRD test with similar performance in a larger setting, we simulated the hypothetical effect on clinical decision making (Materials and methods). Therefore, the following assumptions were made: first, 5.8% of patients potentially benefit from adjuvant chemotherapy [4, 5]. Second, the MRD test's sensitivity for detecting those patients that benefit is equal to the sensitivity for detecting patients who will develop recurrence of disease. This simulation estimated a decrease in benefit of adjuvant chemotherapy in the MRD‐negative group to 3.7% (95% CI: 1.4%–5.6%). On the other hand, in the MRD‐positive group the expected benefit of adjuvant chemotherapy was hypothesised to increase to a median of 9.0% (95% CI: 6.1%–13.3%; Fig. S7).

## 4 Discussion

There is an unmet clinical need to identify stage II–IIIA NSCLC patients who have been successfully cured by surgery alone and will not benefit from adjuvant therapy. Detection of postoperative MRD may help guide adjuvant treatment decisions and reduce overtreatment. Although studies in other types of cancer have demonstrated the ability to detect postoperative MRD [10, 11], and post‐therapy detection of MRD in NSCLC [12, 13], no studies to date have reported postsurgery detection of MRD in stage II–IIIA NSCLC patients with the intent of withholding adjuvant therapy in the MRD‐negative group.

Here we present a combined variant calling and fragment length model to detect postoperative MRD and predict recurrence of disease which reached 75% accuracy in cross‐validation (*P* = 0.0027, log‐rank test). The analyses presented in this work may help drive the integration of various types of information from the same data, ultimately leading to cheaper and more sensitive techniques for detecting postoperative MRD in this setting.

### 4.1 Study design limitations

While the present results are hopeful, they need to be critically nuanced. First and most importantly, this study was designed as an explorative proof‐of‐concept study, and the results should be interpreted as such.

Second, in this study both patients with and without adjuvant chemotherapy were included. Disease recurrence was used as a surrogate endpoint to identify patients with MRD postsurgery. One drawback of this approach is that patients who were cured by adjuvant therapy will show up as false‐positive results in this study design (i.e. MRD‐positive but no recurrence), and skew the model towards more cautious calling of MRD. Since only 14 patients in our cohort started adjuvant chemotherapy, of whom only seven patients completed it, and because of the minimal cure rate of adjuvant chemotherapy, we do not expect this to have a large effect on the results.

Along the same lines, it is important to consider that asking who will develop recurrent disease is not the same as asking who will benefit from adjuvant therapy. By extension, the clinical implications simulated in this study should be interpreted as a best estimate based on the data we have, and not as actual data generated by this study. This estimate might be used to generate hypotheses or inform the design of a follow‐up study.

### 4.2 Model performance

Despite these limitations, this study supplied valuable insights. In order to get an indication of the clinical sensitivity of the combined model we applied it to preoperative plasma samples and patients with nonmalignant disease, using the confirmed presence or absence of a tumour as a clinical gold standard to evaluate the performance of the test. We reached a sensitivity of 75% at 100% specificity, comparable to the performance of other methods that combine mutation detection with fragmentation patterns. For example, in a cohort of 85 stage I–III lung cancer patients Lung‐CLiP reached sensitivities of 54% and 67% in stage II and III respectively, at 98% specificity [24]. MRDetect reached 67% sensitivity for 39 patients with lung adenocarcinoma, of whom 78% with stage I–IIA, at 96% specificity [32]. INVAR reports a sensitivity of 63% in 19 NSCLC patients with stage I–III [25]. DELFI is a different model that uses shallow whole‐genome sequencing combined with artificial intelligence to detect genome‐wide fragmentation patterns [23]. Among 24 stage II–IIIA lung cancer patients, this model reached 96% sensitivity at 80% specificity, and 71% sensitivity at 98% specificity. This indicates that the combined variant detection and fragmentation pattern model developed in this study performs comparably to other state‐of‐the‐art models.

When comparing the performance for detecting MRD, the present model had an accuracy of 75% (95% CI 58%–88%) in cross‐validation, with an NPV of 78% (95% CI 56%–92%). This was comparable to an accuracy of 77% (95% CI 55%–92%) among 22 stage I–III lung adenocarcinoma patients for MRDetect [32], with an NPV of 100% (95% CI 74%–100%). It should be noted that in the MRDetect study only five patients developed recurrent disease, compared to 14 patients in our study, leaving little room for false‐negative results. This is probably caused by a high proportion of stage I disease (14 out of 22 patients), and a comparatively short follow‐up for the negatively tested stage II and III patients in their cohort (*n* = 4). When only considering stage II–III patients in the MRDetect study (*n* = 8) the accuracy was 88% (95% CI 48%–100%) and the NPV 100% (95% CI 40%–100%).

We speculate that while our model shows a highly significant distinction between patients with a high or low risk of developing recurrence (*P* = 0.0029, Fig. 3B), the sensitivity and NPV of our and similar methods will not be sufficient to ethically withhold adjuvant therapy in the clinical application of a postoperative MRD‐test in stage II–III NSCLC. For that reason, the field is working towards increasingly sensitive techniques, and to that end it will be important to obtain as much information as possible from data that is already generated in current and future diagnostic procedures. By combining hybrid capture variant calling data, which can be used for molecular profiling, with fragmentation analyses from the same data, our method is another step in that direction.

Additionally, the postoperative samples in this cohort were obtained relatively soon after surgery (median 6 days). Considering that three out of four false‐positive patients in our cohort had their blood collected within 5 days after surgery and had elevated levels of cfDNA in their blood (Fig. S5), this might indicate a failure of clearance of ctDNA of the primary tumour after surgery. Performance characteristics of the method might be improved by obtaining the blood with a longer interval after surgery to make sure any residual ctDNA from the primary tumour has cleared, although definitive evidence about the optimal timepoint for blood draw after surgery is still lacking [33].

### 4.3 Fragmentation score

To the best of our knowledge the fragmentation score (FS) presented in this work is the first method that derives both a patient‐level and variant‐level fragmentation score from hybrid capture sequencing data. In our model the predictive weight of each fragment is determined by the relative abundance in ctDNA versus nontumour cfDNA. As a consequence, fragments of 130–150 bp and 250–300 bp are given higher predictive weight towards ctDNA, while fragments of 180–210 bp are given higher predictive weight towards healthy cfDNA (Fig. 2A, Fig. S2A).

This method provides several advantages compared to other studies that use fragment length analysis to detect ctDNA. Other models most often define one or several ‘windows’ of fragment lengths that are enriched for ctDNA, such as the window of 100–150 bp [22], 90–150 bp [19] or < 160 bp and 230–310 bp [24]. However, these windows allocate the same predictive weight to each fragment within that window, and the boundaries of the windows may change between different research groups. This is especially detrimental in the 150–160 bp range, which is the most abundant in cfDNA and would have a large impact on the model, even though fragments in that range are abundant in both ctDNA and nontumour cfDNA and therefore poor predictors.

Applying our model to patients with nonmalignant disease, the patient‐level FS was significantly lower than in both preoperative and postoperative patient samples (Fig. 2B). This finding was reproduced in publicly available data of the DELFI cohort, highlighting the reproducibility and broader applicability of the approach (Fig. S3A). The DELFI data were generated from shallow WGS data, confirming that the patient‐level FS performance does not depend on the target area of the sequencing data. However, based on patient‐level FS alone we were unable to reliably distinguish patients with and without recurrence (Fig. S6B), underlining the finding that patient‐level FS is not a silver bullet solution and should be used in conjunction with other means of MRD detection like variant calling.

To reduce the need for BCP‐paired sequencing, methods are needed to distinguish CHIPs from tumour‐derived mutations [34]. To that end we developed a VFS. Since we had access to a rich dataset containing tumour tissue, BCP and plasma sequencing data we were able to report the performance of the VFS on an individual variant level, which has not been reported before. The VFS was capable of distinguishing tumour‐derived variants from nontumour‐derived variants (i.e. CHIPs) with high specificity (84%) and reasonable sensitivity (55%). Sensitivity in variants with at least eight reads improved to 86%, with comparable specificity (82%), at the cost of inconclusive results for 32% of variants (Fig. 3B). Validation of the trained model in a highly characterised public dataset of Grail/MSKCC reached an even superior performance with 82% sensitivity and 92% specificity (Fig. S3B).

Since variants were already classified based on tumour tissue and BCP sequencing, the VFS was not of added value in our current MRD model. We speculate that the VFS could be applied in future studies to filter nontumour‐derived variants with high accuracy, and thereby reduce or eliminate the need to sequence tumour tissue and/or BCP alongside plasma samples.

### 4.4 Clinical implications

In an exploratory hypothesis‐generating simulation we estimated the potential clinical consequences of implementing the MRD prediction model in clinical practice. In the MRD‐negative group we hypothesised that only 3.7% of patients would benefit from adjuvant chemotherapy, potentially tipping the debate towards withholding adjuvant chemotherapy for these patients. However, the simulated data does not correct for chemotherapy undergone by patients in our cohort and represents data from only a small cohort. As such these simulated estimates should be treated as hypothesis generating based on the data we have and not as a prediction of the clinical impact of our model.

At present, adjuvant targeted therapy and immunotherapy are being integrated in early‐stage NSCLC to improve cure rates and long term overall survival [7, 35, 36]. Extensive molecular testing at diagnosis can identify oncogenic drivers and therefore presents an opportunity for targeted treatment in the adjuvant setting. Epidermal growth factor receptor tyrosine kinase inhibitors (EGFR‐TKI) have shown promising efficacy in clinical trials for resected EGFR mutant NSCLC [7]. A plausible future scenario would be the incorporation of precision medicine into treatment of earlier stages of NSCLC. Since this presented MRD model is based on hybrid capture NGS data, this method would provide both a molecular analysis to guide treatment and the identification of MRD as regards which patients would benefit. A recent study with patients who received adjuvant anti‐PD‐1 immunotherapy after melanoma resection showed that nearly half of the patients (43%) developed chronic anti‐PD‐1 related adverse events, defined as persistent symptoms 12 weeks after anti‐PD‐1 discontinuation [37]. Since chronic adverse events can severely impact quality of life in the long term, it will become increasingly important to guide physicians and patients towards informed decisions about adjuvant treatment.

## 5 Conclusion

In conclusion, we present an explorative study to detect postsurgery MRD in stage II–IIIA NSCLC patients, prior to adjuvant therapy. Using only variant calling or only fragment length analysis, we were unable to distinguish patients with or without recurrence of disease with sufficient accuracy. The combined model was capable of stratifying patients after surgery into high versus low risk of developing recurrent disease in a cross‐validation setting. The performance of this model was comparable to other methods that employ combined fragmentation and variant calling. The results of this model could be used as a stepping stone towards a more sensitive model to detect MRD in stage II–IIIA NSCLC patients.

## Conflict of interest

DCLV, MMFS, VvdN, IS, TCL, ML and KLR declare that they have no competing interests. KJH declares an educational grant from Medtronic; KM declares a research grant from AstraZeneca, speakers fee from MSD, Roche, AstraZeneca and Benecke, consultant fee from Pfizer, BMS, Roche, MSD, Abbvie, AstraZeneca, Diaceutics, Lilly, Bayer and Boehringer Ingelheim, and nonfinancial with Roche, Takeda, Pfizer, PGDx and Delfi; MMvdH declares research funding from Bristol Myers Squibb, AstraZeneca, Novartis and Roche/Genentech, consulting or advisory role to Bristol Myers Squibb, AstraZeneca, Pfizer, Merck Sharp & Dohme, Roche/Genentech and Novartis, patents, royalties or other intellectual property with Roche/Genentech, AstraZeneca, Pfizer, Novartis and Merck; DvdB declares a nonpersonal financial competing interest with Roche Diagnostics, where the fees went to the NKI.

## Author contributions

DCLV made substantial contributions to the design of the work, the analysis and interpretation of data, creation of new software, drafted and substantively revised the manuscript. MMFS made substantial contributions to the design of the work, the acquisition and interpretation of data, drafted and substantively revised the manuscript. VvdN made substantial contributions to the analysis and interpretation of data and substantively revised the manuscript. IS made substantial contributions to the acquisition of data and substantively revised the manuscript. TCL, ML and KLR made substantial contributions to the acquisition and analysis of data. KJH made substantial contributions to the conception of the work, interpretation of the data and substantively revised the manuscript. KM and MMvdH made substantial contributions to the conception and design of the work, interpretation of data and substantively revised the manuscript. DvdB made substantial contributions to the conception and design of the work, interpretation of data and drafted and substantively revised the manuscript. All authors read and approved the final version of the manuscript.

## Peer review

The peer review history for this article is available at https://publons.com/publon/10.1002/1878-0261.13267.

## Supporting information


**Fig. S1.** Analytical validation of patient‐level FS.

**Fig. S2.** Fragment‐level fragmentation.

**Fig. S3.** Validation of patient‐level fragmentation score (FS) and variant‐level fragmentation score (VFS) in the DELFI and MSKCC/Grail cohorts respectively.

**Fig. S4.** Technical reproducibility and downsampling of patient‐level FS.

**Fig. S5.** Results of the 20 times repeated 10‐fold cross validation (TTF‐CV).

**Fig. S6.** Kaplan–Meier curves of MRD model using only variant calling, only patient‐level fragmentation score (FS) or variant calling combined with a randomly assigned FS.

**Fig. S7.** Bootstrap simulation of potential clinical implementation.

**Table S1.** Clinical characteristics of the nonmalignant control cases (*n* = 15).

**Table S2.** Variants derived from plasma and tumour tissue samples.

**Table S3.** Bivariate Cox proportional hazard ratio of MRD detection.

## Data Availability

The datasets generated during this study are included in this published article and its supplementary information files are available from the corresponding author on reasonable request. The patient‐level fragmentation score (FS) was validated in the DELFI cohort [22], accessed through FinaleDB [38]. The performance of the VFS was validated in the Grail/MSKCC cohort [31], accessed through EGA data accession number EGAD00001005302 [39].
